# Genomic signal selection analysis reveals genes related to the lambing trait of Hotan sheep

**DOI:** 10.5713/ab.24.0336

**Published:** 2024-11-06

**Authors:** XinKun Wang, Wei Li, QiaoYan Huang, HuiPing Sun, LeXiao Zhu, RuoHuai Gu, Feng Xing

**Affiliations:** 1 Key Laboratory of Tarim Animal Husbandry Science and Technology, Xinjiang Production and Construction Group, Alar, China; 2 College of Animal Science and Technology, Tarim University, Alar, China

**Keywords:** Candidate Genes, Hotan Sheep, Lambing Rate, Selection Signal, Whole-genome Resequencing

## Abstract

**Objective:**

Lambing in ewes is a complex and crucial aspect of sheep production that directly influences economic viability and production efficiency. In the present study, we analyzed the genomes of single lamb (SLE) and twin lamb (TLE) Hotan sheep to elucidate the genetic mechanisms underlying lamb production in Hotan sheep.

**Methods:**

In this study, we used genome-wide resequencing to analyze the genomes of Hotan sheep exhibiting SLE and TLE traits. To identify the population genetic structure and linkage disequilibrium associated with SLE and TLE traits, we employed two complementary genome selection signals: the interpopulation genetic differentiation index (FST) and nucleotide diversity. Subsequently, we performed gene annotation and enrichment analyses of the selected regions of the obtained genome.

**Results:**

Our analysis generated 801 Gb of sequence data, from which 31,864,651 high-quality single nucleotide polymorphic loci were identified. We identified 290 selected regions and 332 genes across the Hotan sheep genome by using two widely adopted selective scanning detection methods (FST statistics and Piratio). Functional annotation and enrichment analysis of these genes identified 13 genes associated with the lambing rate, which were enriched in pathways such as the transforming growth factor-β signaling pathway (*BMPR2*, *ID2*, *SMAD7*, *THBS1*, and *RBX1*), renal cell carcinoma (*PAK1*, *ELOC*), inositol phosphate metabolism (*PLCZ*), non-homologous terminal junction (*RAD50*), ABC transporters (*ABCC4*), and the NET pathway (*H2B*, *H4*, and *H2A*).

**Conclusion:**

This study employed selective elimination analysis to identify candidate genes involved in the regulation of lambing trait in Hotan sheep. By investigating the molecular mechanisms underlying lambing rate in Hotan sheep, we developed molecular markers for twin lambing to enhance reproductive performance and promote the conservation and development of outstanding genetic resources in local Xinjiang sheep.

## INTRODUCTION

Lambing is a major reproductive trait in sheep breeding and production and encompasses numerous biological processes such as hormone secretion, follicular growth, ovulation, fertilization, embryo implantation, placental development, and fetal growth [1]. Genetic variation within the sheep population substantially influences reproductive potential, and maintaining rich genetic diversity (Pi) in the population is usually advantageous for improving reproductive efficiency [2]. Therefore, optimizing the genetic potential and reproductive capacity of sheep is the cornerstone of improving reproductive efficiency.

When a favorable mutation arises, the higher the fitness of the mutant gene, more likely it is to be selected and fixed within the population. This process results in the fixation of the chromosomal region linked to this locus owing to the phenomenon known as the hitchhiking effect, causing a considerable reduction in polymorphism in numerous closely linked chromosomal regions, a pattern referred to as genetic selection analysis [3]. Currently, the population differentiation index (FST) and Pi are widely recognized as effective methods for detecting selective elimination regions, and they can jointly screen strong selection signals of target genes (the top 5% of FST and Pi regions) [4]. For instance, Abdoli et al [5] employed these methods to analyze selection signals in Iranian fat-tailed sheep and identified five genes associated with reproductive traits (*INHBE*, *INHBC*, *TEX12*, *BCO2*, and *WDR70*). Similarly, Guo et al [6] utilized FST and Pi to identify crucial candidate genes affecting the adaptability of Chinese fine wool sheep (*RXPF2*, *EERFC4*, *MSH6*, *PP1R12A*, *THBS1*, *ATP1B2*, *RYR2*, and *PLA2G2E*).

Hotan sheep, a wool-producing breed native to the Hotan region of Xinjiang, China, are renowned for their high heat, disease resistance, and coarse tolerance. Recognizing its importance, the Ministry of Agriculture of China has included Hotan sheep in the List of National Protected Livestock and Poultry Genetic Resources [7]. Despite these advantages, this breed has a relatively low lambing rate, which poses challenges for maximizing farm profits. Increasing the lambing rate is crucial for improving the livelihood of local communities, and is a key objective of sheep breeding. Nonetheless, the low fertility observed in Hotan sheep breeds continues to limit the development of sheep breeding in Xinjiang [8]. Currently, there is limited research on the development and utilization of molecular markers associated with high breeding rates of Hotan sheep. The development of molecular markers is one of the most effective methods for molecular breeding and marker-based selection of molecular correlations. With the advent of high-throughput genotyping technologies and availability of extensive sequence data, the field of labeling technologies has advanced rapidly. Although there is limited information on the reference genome of Hoten sheep, molecular markers remain indispensable for determining genetic variation and species identification with high accuracy and repeatability. Given the variations in the number of lambs per ewe within the Hotan breed, selective elimination analysis was conducted to identify the genes associated with lambing trait. Our study provides a theoretical foundation for improving the reproductive performance of Hotan sheep.

## MATERIALS AND METHODS

### Sample queue and DNA extraction

Hotan sheep, sourced from Hotan Huimin Animal Husbandry Technology Co., Ltd., were chosen as the research subjects, and their reproductive phenotypes, including the number of lambs per ewe, were recorded and statistically analyzed. Based on lambing records, multiparous ewes were categorized into single and twin lamb groups. Blood samples were collected from 180 healthy ewes aged 3 to 4 years (Supplement 1) and preserved at −80°C until further analysis. All experimental animal protocols were performed in accordance with the “Guide to Animal Experimentation” and were approved by the Use Committee under the norms of the Ethics Committee of Tarim University of Science and Technology (SYXK 2020-009). Thirty ewes with single lambs and 30 ewes with twin lambs, along with their corresponding lambing trait records (Supplements 2,3), were used to examine the relationship between single nucleotide polymorphism (SNP) loci and lambing. Genomic DNA was extracted using the standard phenol/chloroform extraction method. DNA integrity was verified using 0.75% agarose gel, and the purity of DNA (OD 260/280 ratio) was determined using a NanoDrop spectrophotometer (Thermo Fisher Scientific, Waltham, MA, USA). The DNA concentration was measured using a Qubit3.0 (Life Technologies, Carlsbad, CA, USA).

### DNA library construction, DNA library inspection, and sequencing

After the DNA fragments passed the test, they were interrupted using a Covaris ultrasonic crusher (Thermo Fisher Scientific) to concentrate the DNA fragments to approximately 200 to 400 bp, and a DNA library was prepared. Subsequently, the construction of the library is completed, the nucleic acid concentration is preliminarily quantified by using Qubit2.0, the library is diluted, and then the inserted fragments of the library are detected using Agilent2100 (Agilent, Beijing, China). When the DNA fragments met expectations, the effective concentration of the DNA library was accurately quantified using quantitative polymerase chain reaction to ensure quality. Arrange computer sequencing for qualified libraries (DNBSEQ): Single-stranded circular DNA molecules replicate by rolling rings, and these DNBs were then sequenced by combined probe-anchored polymerization.

### Quality control, comparison, and identification of single nucleotide polymorphism loci

Fastp software was used for quality control and data filtering. BWA v0.7.17 software package [9] was used to align the filtered clean reads with the reference genome. Samtools v1.11 [10] was used to change the comparison results from SAM files to sorted BAM files, Samtools was used to sort and remove duplicates, and Python v3.9.2 scripts were used to count the comparison rate and coverage. Mutation sites were detected using GATK v4.2.5.0 [11]. All mutation sites were annotated using ANNOVAR (last accessed July 5, 2024) [12]. GATK v4.2.5.0, was used to filter the SNP and insert deletion mutation (INDEL) sites. Subsequently, vcftools v0.1.16 [13] were used to exclude loci with a minor allele frequency (maf) of less than 3% and a genotype deletion rate greater than 20%.

### Population genetic structure analysis

ADMIXTURE v1.3.0 [14] was used to infer the population structure and number of genetic clusters (K) ranging from 2 to 5. Using PLINK v1.9 [15], we performed quality control of the genotype dataset using multiple filter options (−geno: 0.1; −maf: 0.01). Subsequently, we used PHYLIP v3.695 to construct a phylogenetic tree and MEGA v11.0.13 to visualize the kinship data. Principal component analysis (PCA) was conducted using PLINK v1.9 [15] to derive the principal component values for each sample. A scatter plot of principal components was generated using the scatter plot 3d function from the R package to further explore the genetic structure of the population, and the decay of linkage disequilibrium (LD) analysis was performed using PopLDdecay v1.5 software package [16].

### Selective elimination analysis and protein interaction analysis

To identify the selection signal, we used vcftools v0.1.16 to calculate the whole-genome FST and nucleotide diversity (π) using the sliding window method. The parameters used were an FST-window size of 100,000 and FST-window step of 50,000, with each interval between the two populations being 100 kb as the window size and 10 kb as the step size. To minimize false positives, we focused on regions with substantial FST values and extremely low or high θ π ratios (5% left tail and 95% right tail) as candidate genomic regions. These regions were visualized using the R package cmplot.

Furthermore, we annotated gene functions within the selected regions by comparing them with the Gene Ontology (GO) and Kyoto Encyclopedia of Genes and Genomes (KEGG) databases. Based on the literature, genes related to the lambing trait of sheep were screened. To explore protein interactions among the screened genes, protein interaction analysis was performed using STRING. Following these steps, we aimed to identify the genomic regions associated with selection signals, annotate gene functions, and explore protein interactions related to lambing trait in sheep.

## RESULTS

### DNA quality inspection results

**Figure 1:**
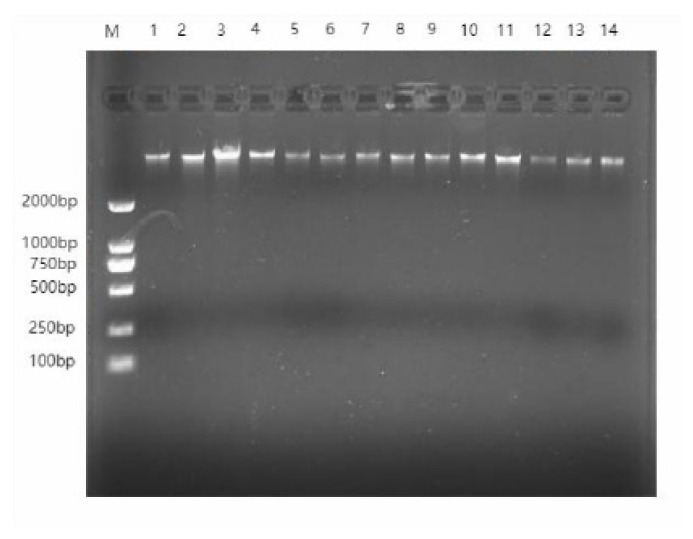
Agarose gel electrophoresis of DNA samples. X axis, gel electrophoresis lane; Y axis, DNA sample position. bp, base pair.

To ensure that 60 DNA samples extracted from Hotan sheep could be used for subsequent database construction, the purity and integrity of DNA samples were detected by 1% agarose gel electrophoresis and ultraviolet spectrophotometry. As shown in Figure 1 (gel electrophoresis results of some DNA samples), the DNA extracted for constructing the Hotan sheep resequencing DNA library has a single bright band, which shows that the DNA integrity is good and can meet the needs of sequencing. The DNA extracted from serum had clear bands, no obvious tailing, and high purity and concentration, and all DNA samples met the requirements of sequencing and database construction.

### Descriptive statistics of animal phenotype

Table 1 presents the phenotypic differences in reproductive characteristics among the various lambing trait of Hotan sheep. Upon analysis of the phenotypic data from Hotan sheep used for blood sample collection, it was observed that the rate of twin lamb (TLE) was notably low, at only 21.8%. Although no substantial differences in pregnancy rates were observed among single lamb (SLE), TLE, and Hotan sheep, variations were noted in estrus onset and cycle duration. TLE Hotan sheep exhibited earlier estrus than SLE Hotan sheep, and the duration of the estrous cycle was longer in TLE Hotan sheep than in SLE and Hotan sheep (Table 1).

### Genome length and genomic data quality

**Figure 2:**
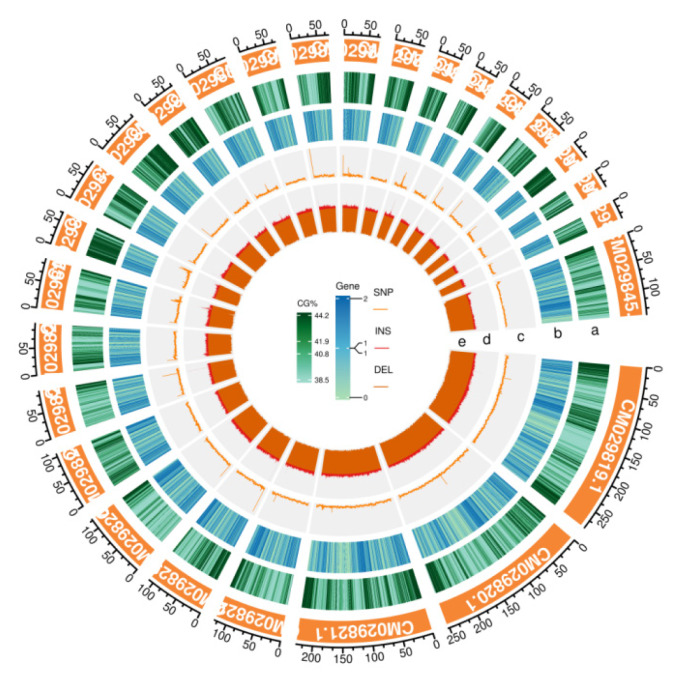
Distribution of whole-genome variation. a, green area indicates GC content; b, the blue area indicates number of genes; c, yellow shading position indicates SNP number; d, rad shading position indicates number of insertions; e, the orange area indicates deletion quantity. GC, guanine-cytosine; SNP, single nucleotide polymorphism.

The reference sheep genomes used in this study are shown in the following table (Table 2). In the present study, 60 Hotan sheep were re-sequenced (Table 3). The data range of the raw data was 90,554,556 to 186,357,182, and the data range of clean reads was 90,551,892 to 186,347,082 after software quality control filtration, with a filtration rate of 99.98% to 99.99%. The sequencing quality was high (Q20≥97.35%, Q30≥ 91.50%). The average cytosine-guanine content was 41.95%, with an average of 1.15 genes in each interval of each chromosome, and an average of 1617 SNP loci was distributed in each interval (Figure 2).

Hotan sheep data were compared with the sheep reference genome using the BWA software Men program, and the comparison rate and average coverage were used as indicators to evaluate data reliability and accuracy. A general comparison of the samples is presented in Table 4. After alignment, the number of reads was between 20.72 and 47.44 GB, the comparison rate of samples was between 99.81% and 99.98%, and the average sequencing depth was 6.77× (4.66×–9.90×), indicating that the database of samples is well established, with an average coverage of 97.70% (94.93% to 98.96%), and indicating a good comparison that could be used for the next step of population genetic structure analysis.

### Analysis of genetic variation information of Hotan sheep

**Figure 3:**
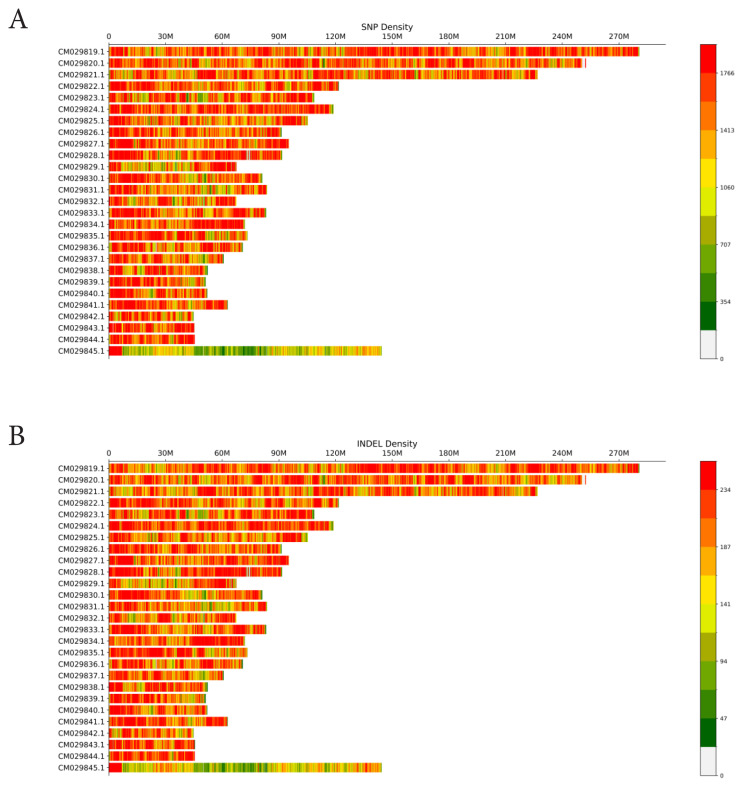
Distribution of SNPs and INDELs in each window. (A) SNP distribution density. X-axis represents chromosome length, Y-axis represents chromosome position, and different colors represent SNP density in each region. (B) INDEL distribution density. X-axis represents chromosome length, Y-axis represents chromosome position, and different colors represent INDEL density in each region. SNP, single nucleotide polymorphism; INDEL, insert deletion mutation.

According to the distribution in the chromosome, we found that the distribution trend of INDEL was consistent with that of SNPs, and both were mostly distributed in intergenic regions. To better understand the distribution of SNPs and InDels in sheep chromosomes better, 100Kb windows were set up, and the density maps of SNPs and InDels in each window were counted. It could be seen that SNPs and InDels had good homogeneity (Figures 3A, 3B).

As can be seen from Table 5, according to the referenced annotation information of the sheep genome, the number of intergenic regions in which variation is annotated to coding genes is the largest, with 31,778,335. Second, the intron region of the coding gene included 16,102,157, with 264,715 mutations in the upstream 1kb region of the coding gene, 255,853 mutations in the downstream 1 kb region of the coding gene, and 5,261 mutations in both the upstream and downstream 1 kb regions of the coding gene. There were 5 mutations in the 5′ untranslated region of the coding gene were 5, and 253,259 mutations in the exon region of the coding gene. There were 1,442 regional variations in the shear sites of the coding genes. There were four mutations that simultaneously existed in the exons and cleavage sites of coding genes.

For the functional annotation of mutations located in exons, the number of synonymous mutation regions was the highest at 130,992. Followed by nonsynonymous mutation regions, including 109,724; the number of frameshift mutation regions was 5,922; The number of frameless mutation regions was 3,241; The number of unknown mutation regions was 2814; The number of termination mutation regions was 2,432; The number of non-terminating mutation regions is 252.

### Population structure analysis

**Figure 4:**
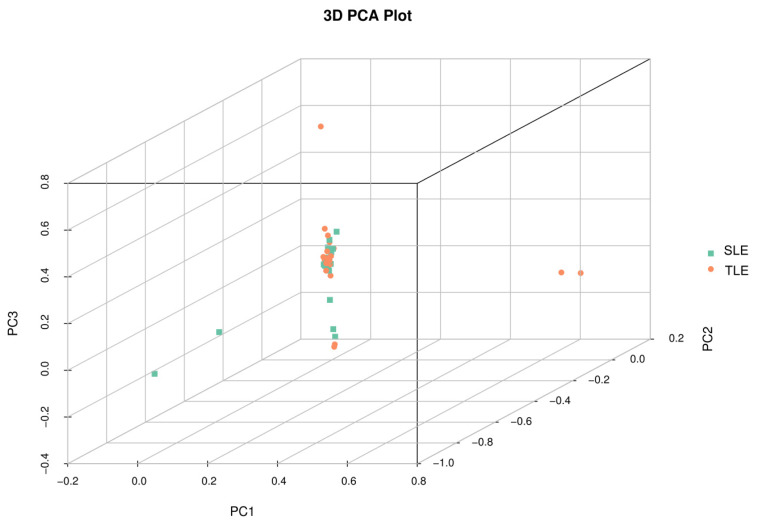
Principal component analysis (PCA) of the SLE and TLE groups. PC1, PC2 and PC3 respectively represent the difference values of the three principal components. SLE, single lamb; TLE, twin lamb.

**Figure 5:**
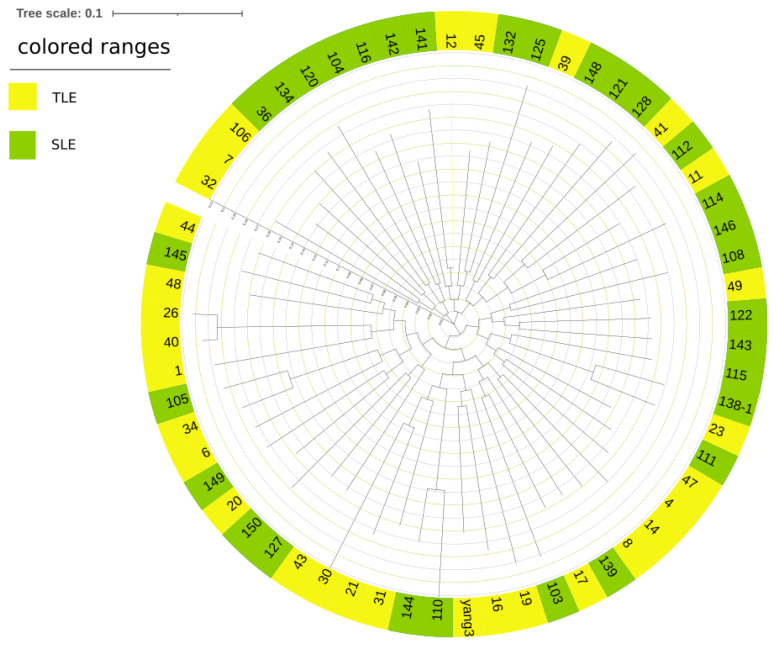
Phylogenetic tree diagram of two Hotan sheep populations. yellow represents TLE Hotan sheep, and green represents SLE Hotan sheep. TLE, twin lamb; SLE, single lamb.

PCA analysis based on the SNPs in autosomes (Figure 4) showed that most SLE and TLE groups were not obviously separated, and only a few groups were scattered in the opposite direction. Considering the existence of outliers, a few subgroups may not represent the whole sample, and these samples may not have an obvious structure. The results of the adjacent phylogenetic trees are shown in Figure 5. Geographically, the individual compositions of the two Hotan sheep populations are not clear.

**Figure 6:**
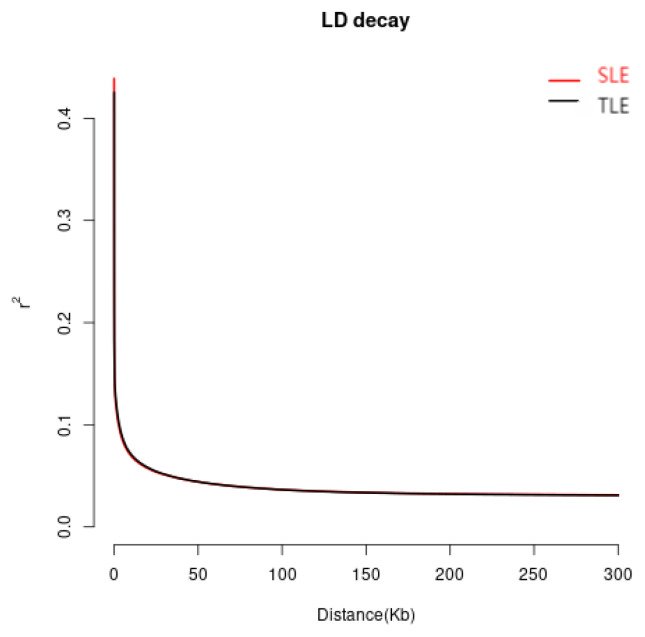
LD attenuation diagram. The X-axis represents the attenuation distance, and the Y-axis represents the LD level of the population. LD, linkage disequilibrium; SLE, single lamb; TLE, twin lamb.

To detect LD attenuation in Hotan sheep, the r2 values of the SLE and TLE groups were calculated. The results of the LD attenuation in this experiment are shown in Figure 6. These findings indicate that the SNP distance between the SLE and TLE groups in Hetian sheep decreased rapidly in the range of 0 to 50 kb, with r2 reducing from 0.4 to 0.1, and the LD attenuation rate between the two groups was nearly identical. The decay rates of Hetian sheep with different litter sizes were very similar, which suggests that differentiation was not considerable. This may be attributed to individuals originating from large populations with similar origins, and not being subjected to varying degrees of (artificial or natural) selection.

**Figure 7:**
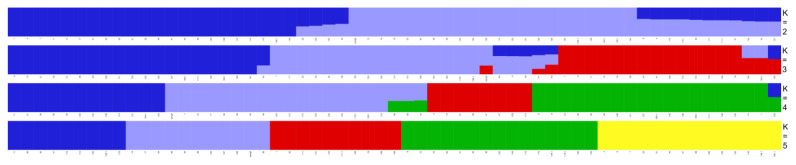
Population structure of 60 individuals using ADMIXTURE with k = 2 to 5. Each color represents a subgroup, the x-axis represents the sample name, and the y-axis represents the k value.

Structural analysis showed that the Cross-Validation error was the smallest when K = 2, and SLE and TLE began to separate when K = 3. As the k value decreased, the separation gradually became obvious, and the SLE and TLE were distinctly separated when K = 2 (Figure 7).

### Selective elimination analysis

**Figure 8:**
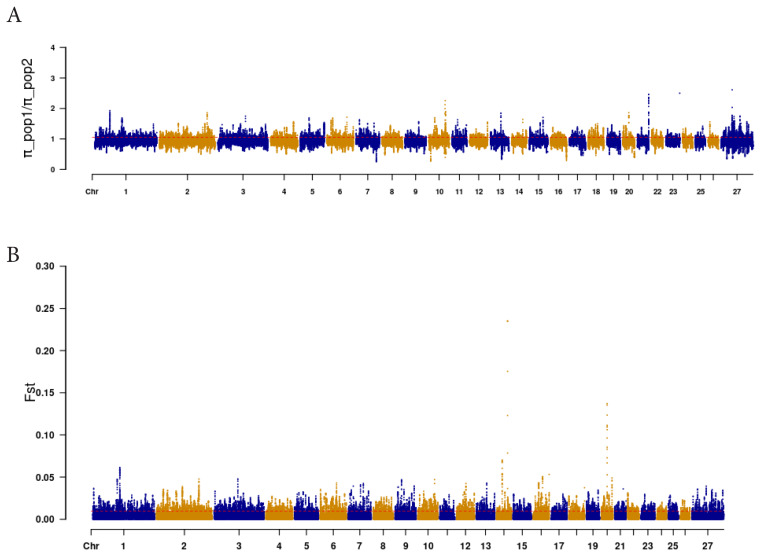
FST and PI analysis of Manhattan plot. (A) Manhattan diagram of population genetic differentiation index analysis of SLE and TLE. The X-axis represents chromosome position and the Y-axis represents FST value. (B) Manhattan diagram of SLE and TLE nucleic acid diversity analysis. The x-axis represents chromosome position and the y-axis represents pi value. The top 0.05% of the empirical distribution of FST and 0.05% of the PI scores are indicated by the dotted lines. FST, differentiation index; PI, nucleotide diversity; SLE, single lamb; TLE, twin lamb; pi, genetic diversity.

**Figure 9:**
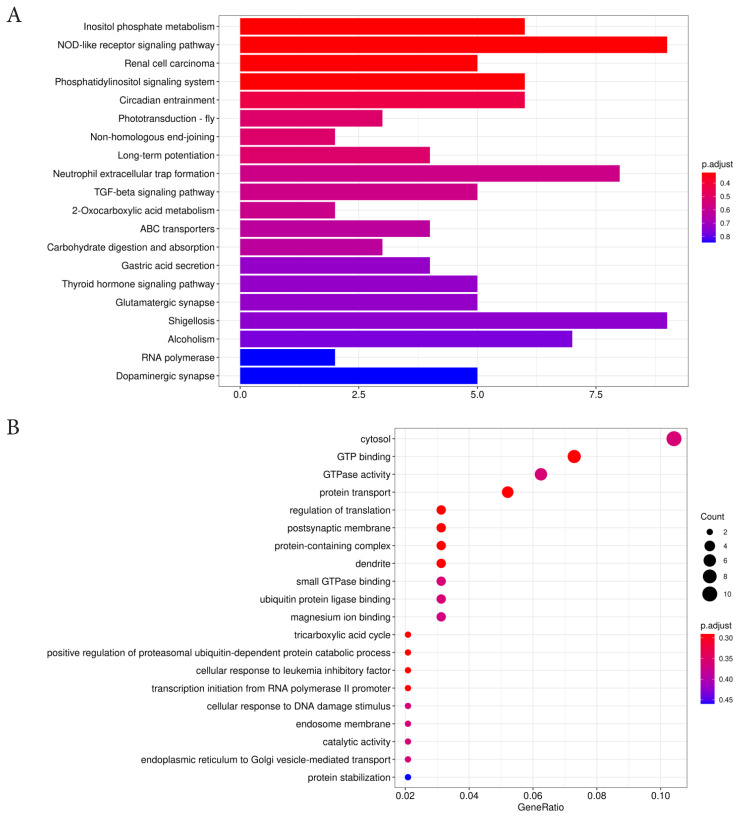
GO and KEGG gene distribution map. (A) KEGG pathway enrichment analysis of the candidate genes in Hotan sheep. The X-axis represents the number of enriched genes and the Y-axis represents the enriched items. (B) GO analysis of candidate genes of Hotan ewes. The size of the filled circles indicates the number of DEGs involved. The size of the scattered dots represents the number of genes, whereas the color represents the contribution of a specific gene set in the pathway to the total gene set. TGF-β, transforming growth factor-β; GO, gene ontology; KEGG, Kyoto encyclopedia of genes and genomes; DEG, differentially expressed genes.

**Figure 10:**
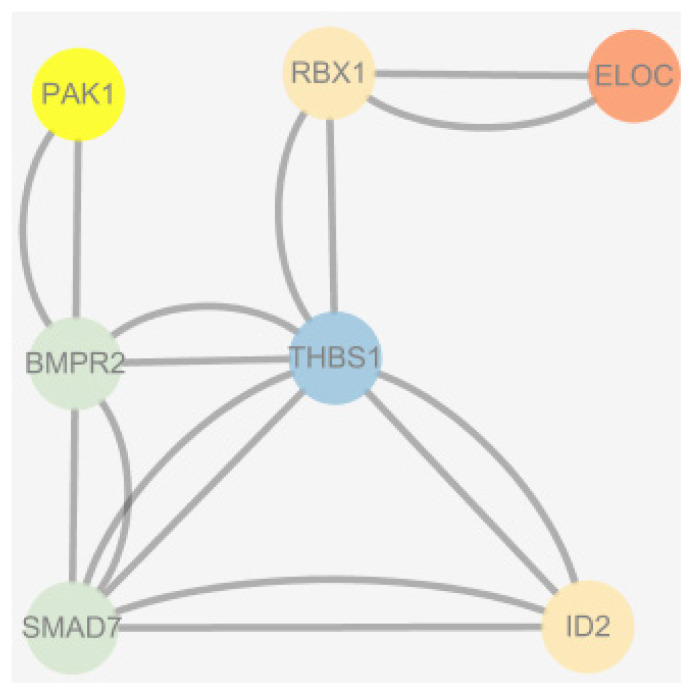
Protein interaction map of the screened genes. The chart shows the correlation between each gene and the interactions between them.

Based on selective elimination analysis (Figure 8), we analyzed the common areas of FST and Pi rates in SLE and TLE, wherein the regions above the threshold line (top 0.05%) were considered. Subsequently, 332 genes were annotated within these areas (Supplement 4). The GO and KEGG pathway analysis results are shown in Figure 9. Upon review of relevant references for 332 genes, it was found that 13 genes (*BMPR2*, *ID2*, *SMAD7*, *PLCZ*, *PAK1*, *ELOC*, *RAD50*, *H2A*, *H2B*, *THBS1*, *RBX1*, *H4*, and *ABCC4*) were associated with litter size. These genes were enriched in the TGF-β signaling pathway, renal cell carcinoma, inositol phosphate metabolism, non-homologous terminal junction, ATP-binding cassette (ABC) transporters, and NET pathway. Furthermore, protein interaction analysis revealed interactions among *BMPR2*, *ID2*, *SMAD7*, *PAK1*, *ELOC*, *RAD50*, *THBS1*, and *RBX1* (Figure 10), providing an in-depth understanding of the interrelationship of these genes in the context of the regulation of lambing trait in Hotan sheep.

## DISCUSSION

### Genetic diversity and population structure

Population Pi can accurately analyze the ancestral group and individual lineage composition of livestock and poultry populations. Techniques such as PCA, genetic map analysis, and population genetic structure analysis can provide comprehensive insights, and the results can be verified. Such analysis is crucial for the effective utilization and protection of population genetic resources. In this study, the population structure of Hotan sheep with different numbers of lambs and Tian sheep was studied. It was found that the population structures of the two groups were not completely separated, and a small number of sheep individuals were genetically mixed, which may be because some individuals in the two groups had the same genome ancestor. For example, the phenotype of the individual “105” is the low fertility group, however, the results of population genetic structure show that it is a high fertility group, and the experimental results are similar to those of Wang et al [17]. Considering that the ewes sampled had the same feeding environment and management mode, it may be due to individual or environmental factors that ewes with a high reproductive genetic basis have fewer lambs. The characteristics of natural selection events and reproductive behavior experienced by different species or groups can be reflected through a linkage imbalance. Domestication and selection will lead to a decline in population Pi and strengthening of linkage between loci. The higher the degree of domestication, the greater the selection intensity and the lower the LD decay rate of the population. However, the attenuation curves of the SLE and TLE groups were similar, the attenuation speed was fast, and the attenuation distance was almost the same, which indicates that the degree of domestication of the Hetian sheep population was low, and the selection intensity was small. The above results show that there is a certain degree of population genetic differentiation between the SLE and TLE groups, but the degree of differentiation is small, which may be from the same population in the same sheep farm or the population has not been strongly artificially selected, which is in line with the actual situation.

### Analysis of selected genes in different litter size groups of Hotan sheep

Selection elimination analysis is primarily used to identify the genes selected during domestication and those selected during the adaptive evolution of species. The lambing trait, a quantitative trait regulated by multiple genes and factors, has been subjected to positive selection to a certain extent during the evolution and development of sheep. In this study, Hotan sheep of the same population with different numbers of lambs were selected for selection elimination analysis to screen for candidate genes related to the lambing trait. The FST values of the high- and low fertility groups were calculated, the Fst values were corrected to ZFst values, and the Pi values were calculated using the sliding window method with a window size of 100 kb and step size of 10 kb. Subsequently, the θ π ratio (pi.SLE.windowed.pi/pi.TLE.windowed.pi) was calculated, and the common window of the joint analysis of ZFst and θ π ratio was selected as the candidate window. Setting the top 5% as the threshold, windows with both the top 5% ZFst and θ π values were designated as candidate regions, ultimately yielding 290 shared genomic regions. From these 290 regions, 332 candidate genes were identified by annotation. These genes are primarily concentrated in TGF-β, renal cell carcinoma, phosphoinositide metabolism, non-homologous terminal junction, ABC transporter, and the NETs pathway. The association of PAK1 with the Hotan sheep lambing trait is further supported by a study on Qira Black sheep [18], which also suggested a possible influence of *PAK1* on precocious puberty onset.

### Correlation genes of lambing trait found by analysis

Among the pathways detected in this study, transforming growth factor-β (TGF-β) was the most closely related to the lambing trait. TGF-β is a pleiotropic cytokine that regulates many cellular processes, such as cell growth, differentiation, apoptosis, migration, cell adhesion, and immune response [19]. TGF-β family ligands are intricately related to the control of ovulation and fertilization and the establishment and maintenance of pregnancy. In mammals, even the earliest stages of reproductive development, including the specification of male and female species, are controlled by TGF-β-related proteins [20]. *SMAD7* has been proven to play a role as an intracellular antagonist of TGF-β family signaling, which is a key regulator of TGF-β bone morphogenetic protein (BMP) signaling through a negative feedback loop, and inhibits TGF-β signaling by competing with receptor-regulated SMADs, which are involved in oocyte-somatic cell interaction and regulation of granulosa cell function [21]. Gao et al [22] regulated the expression level of *SMAD7* in ovarian granulosa cells by overexpression and small interfering RNA knockdown, thus affecting follicular development in mice. *SMAD7* signal transduction can reduce the dependence of TGF-β on *ID2* [23]. As the inhibitor of the DNA binding (ID) gene is the main target of BMP/SMAD signal transduction, downregulation of *ID2* leads to the inhibition of TGF-β. da Silveira et al [24] and others screened exosomes isolated from mares’ follicles in the middle estrus and preovulatory periods and identified microRNA (miRNA)s that are expected to regulate TGF-β/BMP signal members. *ID2*, a predicted target of exosome miRNAs, was found to exist in granulosa cells and exosomes in the follicular fluid of the middle estrus and preovulatory periods. Simultaneously, *BMPR2*, the receptor for *BMP4*, regulates germ cell activity and activates *ID2* [25]. *BMPR2* is a type II receptor of the TGF-β family that exhibits serine/threonine protein kinase activity. *BMPR2* can be detected at all stages of follicular development and plays an important role in promoting sex hormone secretion, ovarian development, follicular growth, and granulosa cell proliferation. Studies have shown that *BMPR2* plays an important role in the growth and function of mammalian and chicken follicles [26]. *THBS1* is an important activator of TGF-β, which can change the conformation of the TGF-β protein, expose its binding site with cell receptors, and activate the TGF-β signaling pathway [27]. Overexpression of *THBS1* substantially activates the TGF-β pathway, and the TGF-β signaling pathway can be inhibited by down-regulating *THBS1*. At the same time, the expression of *THBS1* in fibroblasts increases, promoting the growth and migration of fibroblasts and TGF-β expression [28].

TGF-β inhibits the expression of *RAD50* and stimulates TGF-β expression in cultured fetal ovarian fibroblasts [29]. *RAD50* not only plays a role in the repair of double-stranded breaks and homologous synapses during female meiosis but also affects the elimination of oocytes that have not been repaired after birth. This suggests that similar to spermatocytes, it may play a role in monitoring meiotic recombination during the prophase of meiosis. Knockdown of *RAD50* leads to a decrease in the expression level of two proteins in spermatocytes (GC-2spd), and downregulation of *RAD50* inhibits the proliferation of GC-2spd cells and promotes apoptosis, which is consistent with the simultaneous decrease in *RAD50* in the testes of spermatogenic failure patients [30]. Changes in the expression of RAD50 in the testis may lead to an imbalance in male germ cell proliferation and apoptosis, ultimately damaging spermatogenesis. This also indicated that *RAD50* may play a key role in the repair, proliferation, differentiation, and apoptosis of male germ cells. P21-activated kinase 1(*PAK1*) is an effector of the RhoGTP enzymes RAC1 and CDC42, which are involved in regulating mitotic events such as chromatin condensation and subsequent chromosome capture, movement, and separation. High expression of *PAK1* inhibits TGF-β function [31]. Previous reports have shown that *PAK1* is a key regulator of various neuronal processes and binds to the BMPI receptor, ALK2 [32]. Sperm-specific phospholipase Czeta (*PLCz*) is a candidate sperm-derived oocyte-activating factor that can trigger a series of characteristic physiological stimulation fertilization processes through cytoplasmic Ca2+ [33]. *PLCz* is expressed in the epididymis, acrosome of sperm, equatorial segment, head-middle junction, and the main part of the flagella. Some evidence suggests that sperm deliver *PLCz* when they fuse with oocytes. It has been reported that *PLCz* can penetrate the oocyte in the sperm acrosome, which is helpful for successful fertilization [34], and is the key protein in the process of sperm egg fusion in mammals. *ABCC4* is mainly localized in the uterine cavity epithelial and glandular epithelial cells, and is expressed in the endometrium in a pregnancy and stage-dependent manner. *ABCC4* plays a key role in supporting the establishment and maintenance of pregnancy by regulating prostaglandin E2 transport in the mother and fetus. Studies have shown that *ABCC4* is expressed in fertilized eggs of pigs during early pregnancy and that *ABCC4* may act on the endometrium and fertilize eggs in an autocrine or paracrine manner [35], thus promoting the establishment and maintenance of pig pregnancy. *RBX1* plays a role in E3 ligase complexes, which regulate various biological processes including DNA repair and mitotic fidelity. During nucleotide excision and repair, histone *H2A* ubiquitination can repair damage regulated by *RBX1* [36]. *RBX1* is also an important part of the SCF complex, and its subcellular localization indicates that *RBX1* plays an important role in the meiosis and maturation of mouse oocytes [37]. An imbalance in *RBX1* leads to late stagnation and maintains cells in the germinal vesicle (GV) stage. *RBX1* can bypass oocytes, the GV stage, and metaphase MII (II) stage.

Core histones are composed of four proteins (*H2A*, *H2B*, *H3*, and *H4*), and the study of histone modification modes is important to understand their functions in biological processes. *H2A* and *H4* are conserved in the nucleus of mature mouse sperm, indicating that a certain amount of *H2A* and *H4* conserved in the nucleus may represent a more accurate marker for mature mouse sperm [38]. In the prophase of meiosis, all *H4* modifications are very high, and elongated sperm cells exhibit acetylation, which increases histone *H4*. Modification of the terminal tail of histone H4N changes considerably in male germ cells during spermatogenesis, suggesting that modification of histone *H4* plays an important role during this stage of spermatogenesis. H2A-H2B dimer is located on both sides of the nucleosome, stabilizing the entire structure of the nucleosome and regulating its dynamics such as DNA unpacking and sliding. This epigenetic regulation is achieved through post-translational modifications and the incorporation of histone variants [39]. The bending dynamics of H2A-H2B dimers are regulated by the interaction of the H3-H4 tetramer, distortion defects in nucleosome DNA, and amino acid sequences of histones. H2A-H2B modified fast short-term memory is helpful for H3-H4 chromatin to return to a stable state. We propose that the short-term guidance provided by H2A-H2B in the process of DNA replication is based on the long-term maintenance of H3-H4 and that the exchange rate of H3A-H4 before and after H2A-H2B replication is higher [40].

## CONCLUSION

In this study, we analyzed the lambing trait of Hotan sheep of different litter sizes. Although the two populations representing TLE and SLE exhibited some degree of genetic differentiation, their overall genetic background was similar. Through selective signal elimination analysis, we identified 13 candidate genes that might be associated with lambing trait: *BMPR2*, *ID2*, *SMAD7*, *PLCZ*, *PAK1*, *ELOC*, *RAD50*, *H2A*, *H2B*, *THBS1*, *RBX1*, *H4*, and *ABCC4*. Functional enrichment analysis of these genes showed that the TGF-β signaling pathway, renal cell carcinoma, non-homologous terminal junction, NETs pathway, ABC transporter, and other pathways may be involved in the regulation of lambing trait in Hotan sheep, thus influencing the fertility of this population.

## Figures and Tables

**Table 1:** Analysis of phenotypic data for single and multiple Hotan lambs

Items	Sample size	Puberty (months old)	Erotic cycle (week)	Pregnancy (month)
SLE	30	8.37	15.73	5
TLE	30	8.33	15.43	5

SLE, single lamb; TLE, twin lamb.

**Table 2:** Sheep reference genome information table

Sequence number	Total length	GC content(%)	Gap rate(%)	N50 length	N90 length
58	2,653,843,355	41.946	0.185	105,184,753	52,181,980

GC, guanine-cytosine.

**Table 3:** General situation of quality of individual weight sequencing data of Hotan sheep

Sample	Raw reads	Clean reads	Reads passed	Q20_ratio (%)	Q30_ratio (%)	GC content (%)
141	98035694	98023516	98023516	97.47	91.60	43.68
39	109274732	109270698	109270698	97.70	92.63	43.19
110	99750450	99743280	99743280	97.61	92.16	43.84
41	97438834	97436380	97436380	97.64	92.46	43.93
20	138656928	138653726	138653726	97.82	93.01	42.64
12	130095278	130092914	130092914	97.75	92.82	43.31
6	125015778	125011928	125011928	97.75	92.80	43.16
49	129982496	129979320	129979320	97.83	93.05	43.01
34	132904874	132902100	132902100	97.79	92.92	42.74
31	142910952	142906396	142906396	97.76	92.83	43.11
21	121361122	121357606	121357606	97.67	92.53	42.91
23	146897010	146894200	146894200	98.04	93.72	43.49
45	145263554	145260238	145260238	98.00	93.57	43.25
48	156868286	156864632	156864632	97.96	93.45	42.99
44	157786980	157783412	157783412	98.02	93.63	42.92
120	93925584	93917596	93917596	97.43	91.51	44.06
26	101043054	101033180	101033180	97.51	91.82	43.77
125	99921596	99913470	99913470	97.58	92.05	44.22
14	113615934	113614080	113614080	97.73	92.73	42.81
30	90554556	90551892	90551892	97.37	91.65	43.73
134	104189536	104181988	104181988	97.59	92.14	43.72
1	115178384	115174358	115174358	97.42	91.77	43.79
32	120738408	120734910	120734910	97.65	92.48	43.78
40	115880716	115878606	115878606	97.56	92.22	42.72
11	121378948	121376168	121376168	98.08	93.83	42.91
8	141670382	141667948	141667948	98.05	93.73	43.15
43	136956128	136953254	136953254	97.70	92.67	42.63
47	151064716	151061394	151061394	97.92	93.31	42.94
4	148975954	148973398	148973398	97.78	92.86	42.81
yang3	161038708	161035892	161035892	97.96	93.44	42.88
105	104898826	104894726	104894726	97.75	92.91	43.07
19	118874796	118870548	118870548	97.59	92.28	43.14
16	128938770	128936186	128936186	97.81	92.99	42.99
36	98059024	98055844	98055844	97.35	91.57	43.27
128	103151332	103147416	103147416	97.54	92.13	43.87
17	141486574	141482494	141482494	97.85	93.13	42.93
148	94964462	94945508	94945508	97.91	93.40	43.17
116	93037908	93032796	93032796	97.67	92.69	42.93
112	135971260	135931766	135931766	97.79	92.92	42.61
127	154111280	154094160	154094160	98.01	93.58	42.97
115	154734360	154718432	154718432	98.22	94.25	42.63
144	144969718	144961370	144961370	98.44	94.50	42.82
150	99689814	99671594	99671594	97.88	93.02	41.72
108	105917946	105908700	105908700	97.86	93.26	42.90
143	116226176	116201898	116201898	97.44	91.84	42.59
145	162695580	162688698	162688698	98.26	94.37	42.10
106	158605530	158584126	158584126	97.98	93.51	42.80
7	164468502	164427702	164427702	98.18	94.11	42.78
114	114945978	114911920	114911920	98.20	94.19	42.87
103	97650300	97642952	97642952	98.19	94.26	42.08
111	186357182	186347082	186347082	98.25	94.35	42.60
104	101054062	101035468	101035468	97.39	91.54	41.56
149	97192490	97180160	97180160	97.44	91.50	41.50
121	129389118	129345512	129345512	97.81	92.99	42.98
122	139566900	139528770	139528770	98.07	93.79	42.57
139	164795690	164762256	164762256	98.02	93.63	43.14
138-1	93448986	93446568	93446568	97.35	91.69	43.06
146	113260928	113250466	113250466	97.42	91.90	43.08
142	150709944	150698946	150698946	98.02	93.65	42.58

GC, guanine-cytosine.

**Table 4:** Statistical survey of sequencing depth and coverage of Hotan sheep

Sample	Mapped reads (bp)	Total reads (bp)	Mapping rate (%)	Average depth (X)	Coverage (%)
60	120.11±45.42	120.04±15.40	99.89±0.08	6.95±2.01	7.18±2.6

bp, base pair.

**Table 5:** Notes on genetic variation information of Hotan sheep population

Annotation_position	Variant_number	Variant_type	Variant_number
Intergenic	31778335	Nonsynonymous	109724
Upstream	264715	Synonymous	130992
Exonic	253259	Unknown	2814
Intronic	16102157	Frameshift	5922
Downstream	255853	Nonframeshift	3241
Upstream;Downstream	5261	Stopgain	2432
Splicing	1442	Stoploss	252
UTR5	5		
Exonic;Splicing	4		

## SUPPLEMENTARY MATERIAL

**Supplement 1**. Phenotypic data of Hotan sheep

**Supplement 2**. Lamb production records of Hotan sheep with SLE

**Supplement 3**. Lamb production records of Hotan sheep with TLE

**Supplement 4**. Genes screened based on selective elimination analysis
