# Supplementary material for: Genomic signal selection analysis reveals genes related to the lambing trait of Hotan sheep
Source: Anim Biosci. 2024 Nov 6;38(7):1384–97. doi: 10.5713/ab.24.0336 (PMC12229938; doi:10.5713/ab.24.0336)
Supplement: Supplementary file 1 [file ab-24-0336-Supplementary-1.pdf]

S1 Phenotypic data of Hotan sheep

Ear number	puberty	Number of lamb	SLE and TLE	Erotic cycle	Pregnancy
00226722	8	1	SLE	14	5
00226742	8	1	SLE	14	5
01270758	8	1	SLE	15	5
00226740	8	1	SLE	14	5
00846120	8	1	SLE	16	5
01270726	8	1	SLE	15	5
0226717M	8	1	SLE	15	5
00226701	8	1	SLE	15	5
01270754	8	1	SLE	16	5
00226707	8	1	SLE	14	5
00226736	9	1	SLE	15	5
00226718	9	1	SLE	17	5
01270720	9	1	SLE	13	5
00226768	8	1	SLE	15	5
01215472	8	1	SLE	14	5
00226779	9	1	SLE	14	5
00226778	8	1	SLE	16	5
00281396	8	1	SLE	16	5
01270751	8	1	SLE	15	5
01270712	9	1	SLE	15	5
00226706	8	1	SLE	15	5
00226747	8	1	SLE	14	5
HT15464	8	1	SLE	15	5
00226737	8	1	SLE	14	5
01270711	9	1	SLE	16	5
00226786	9	1	SLE	16	5
01270739	9	1	SLE	17	5
00226765	9	1	SLE	16	5
00842020	9	1	SLE	13	5
00226738	9	1	SLE	13	5
00226788	9	1	SLE	15	5
00584947	8	1	SLE	14	5
01270730	8	1	SLE	13	5
00226799	8	1	SLE	16	5
00226724	8	1	SLE	17	5
01270722	8	1	SLE	14	5
00226749	8	1	SLE	14	5
01270736	8	1	SLE	15	5

00226733	9	1	SLE	16	5
00226715	9	1	SLE	15	5
01270746	9	1	SLE	15	5
00226704	8	1	SLE	15	5
HT15485	8	1	SLE	15	5
01270748	8	1	SLE	16	5
01270742	8	1	SLE	16	5
00226746	8	1	SLE	16	5
01270750	9	1	SLE	16	5
01270749	9	1	SLE	16	5
00226789	8	1	SLE	15	5
00270729	8	1	SLE	15	5
01270727	8	1	SLE	18	5
00226757	8	1	SLE	17	5
00226732	9	1	SLE	15	5
01282321	9	1	SLE	15	5
01270745	9	1	SLE	16	5
01270759	8	1	SLE	15	5
01270743	8	1	SLE	16	5
00226760	8	1	SLE	15	5
01270752	8	1	SLE	15	5
00226782	9	1	SLE	15	5
00748584	9	1	SLE	15	5
00226762	8	1	SLE	16	5
01270723	9	1	SLE	17	5
00226735	9	1	SLE	14	5
01270732	8	1	SLE	14	5
00226777	8	2	TLE	15	5
01270719	8	2	TLE	16	5
01270737	8	2	TLE	15	5
00226716	8	2	TLE	16	5
01270744	8	2	TLE	17	5
00226703	8	2	TLE	14	5
01270715	8	2	TLE	15	5
01270747	8	2	TLE	16	5
00449764	8	2	TLE	15	5
01270741	8	2	TLE	16	5
01270760	8	2	TLE	15	5
01270731	8	2	TLE	16	5
01270728	9	2	TLE	15	5
00226725	8	2	TLE	17	5
00226705	8	2	TLE	14	5

01270734	8	2	TLE	15	5
01270740	8	2	TLE	17	5
00226780	8	2	TLE	16	5
00226769	9	2	TLE	15	5
01270724	9	2	TLE	15	5
00226751M	9	2	TLE	16	5
00226710	9	2	TLE	15	5
01270721	9	1	SLE	14	5
00226775	8	1	SLE	14	5
00226720	8	1	SLE	17	5
01270753	8	1	SLE	14	5
01270714	8	1	SLE	14	5
01270718	8	1	SLE	15	5
00226731	8	2	TLE	16	5
01270713	8	1	SLE	14	5
00226724	8	1	SLE	16	5
01270738	8	1	SLE	15	5
01270716	8	2	TLE	15	5
00226772	8	1	SLE	15	5
01270735	8	1	SLE	15	5
01270757	9	2	TLE	15	5
00226756	9	2	TLE	16	5
01270725	9	2	TLE	17	5
00226741	9	1	SLE	17	5
00226744	9	1	SLE	17	5
00226711	8	1	SLE	17	5
01270756	9	1	SLE	17	5
00226726	9	1	SLE	18	5
00226793	9	1	SLE	16	5
00226787	9	1	SLE	16	5
01270717	8	1	SLE	16	5
01270755	8	1	SLE	14	5
00226737	8	1	SLE	14	5
00226785	8	1	SLE	15	5
00226708	8	1	SLE	16	5
00226700	8	1	SLE	17	5
00226833	8	1	SLE	16	5
00226864	8	1	SLE	17	5
00226890	8	1	SLE	13	5
00226783	9	1	SLE	15	5
00226770	9	1	SLE	14	5
00226714	9	1	SLE	14	5

00226735	8	1	SLE	14	5
00226795	9	1	SLE	14	5
00226784	8	1	SLE	17	5
00226767	8	1	SLE	17	5
00226776	8	1	SLE	17	5
00226784	8	1	SLE	17	5
00226730	9	1	SLE	17	5
00226739	9	1	SLE	15	5
00226759	9	1	SLE	15	5
00226774	8	1	SLE	15	5
00226791	8	1	SLE	13	5
00226712M	8	1	SLE	13	5
00226713	8	1	SLE	18	5
00226719	8	1	SLE	18	5
00226796	8	1	SLE	13	5
00226723	8	1	SLE	13	5
00226745	9	1	SLE	14	5
00226761	9	1	SLE	14	5
00226752M	8	1	SLE	14	5
00226753	8	1	SLE	13	5
00226766	8	1	SLE	13	5
01226702	8	1	SLE	13	5
21016301	8	1	SLE	16	5
3022063	8	1	SLE	14	5
00320015	8	1	SLE	15	5
00153101	9	1	SLE	15	5
3022001	9	1	SLE	15	5
0053105	9	1	SLE	14	5
070026	8	1	SLE	14	5
120156	8	1	SLE	14	5
T036014	8	2	TLE	14	5
0300784	8	1	SLE	14	5
106471	8	1	SLE	14	5
066017	9	1	SLE	15	5
007011	8	1	SLE	15	5
826043	8	1	SLE	15	5
94821104	8	1	SLE	15	5
8260119	8	2	TLE	14	5
6990114	9	1	SLE	16	5
120156	9	2	TLE	16	5
468206	9	2	TLE	14	5
826073	8	2	TLE	14	5

066017	8	2	TLE	16	5
T036013	8	1	SLE	16	5
0026701	8	2	TLE	14	5
0127084	8	1	SLE	14	5
826011	8	1	SLE	15	5
2191483	9	2	TLE	15	5
2191487	8	2	TLE	15	5
3200139	8	2	TLE	15	5
067018	8	2	TLE	15	5
00153103	9	2	TLE	15	5
0127436	8	1	SLE	15	5
0368482	8	1	SLE	14	5
066087	8	1	SLE	14	5
HT0465	8	1	SLE	15	5
012701	9	1	SLE	14	5
