# Supplementary material for: Genomic signal selection analysis reveals genes related to the lambing trait of Hotan sheep
Source: Anim Biosci. 2024 Nov 6;38(7):1384–97. doi: 10.5713/ab.24.0336 (PMC12229938; doi:10.5713/ab.24.0336)
Supplement: Supplementary file 2 [file ab-24-0336-Supplementary-2.pdf]

S2 Lamb production records of Hotan sheep with SLE

Ear number	puberty	Number of lamb	SLE and TLE	Erotic cycle	Pregnancy
1270742	8	1	SLE	16	5
1270750	9	1	SLE	16	5
226789	8	1	SLE	15	5
270729	8	1	SLE	15	5
1270727	8	1	SLE	18	5
226757	8	1	SLE	17	5
226732	9	1	SLE	15	5
1270745	9	1	SLE	16	5
1270743	8	1	SLE	16	5
1270723	9	1	SLE	17	5
226762	8	1	SLE	16	5
226735	9	1	SLE	14	5
1270721	9	1	SLE	14	5
226775	8	1	SLE	14	5
1270714	8	1	SLE	14	5
1270718	8	1	SLE	15	5
226731	8	1	SLE	16	5
1270724	8	1	SLE	16	5
1270738	8	1	SLE	15	5
1270735	8	1	SLE	15	5
226711	8	1	SLE	17	5
1270756	9	1	SLE	17	5
226726	9	1	SLE	18	5
226793	9	1	SLE	16	5
226735	8	1	SLE	14	5
226700	8	1	SLE	17	5
226833	8	1	SLE	16	5
226784	8	1	SLE	17	5
226739	9	1	SLE	15	5
22759	9	1	SLE	15	5
