# Supplementary material for: Genomic signal selection analysis reveals genes related to the lambing trait of Hotan sheep
Source: Anim Biosci. 2024 Nov 6;38(7):1384–97. doi: 10.5713/ab.24.0336 (PMC12229938; doi:10.5713/ab.24.0336)
Supplement: Supplementary file 3 [file ab-24-0336-Supplementary-3.pdf]

S3 Lamb production records of Hotan sheep with TLE

Ear number	puberty	Number of lamb	SLE and TLE	Erotic cycle	Pregnancy
226777	8	2	TLE	15	5
1270719	8	2	TLE	16	5
12270737	8	2	TLE	15	5
226716	8	2	TLE	16	5
1270744	8	2	TLE	17	5
226703	8	2	TLE	14	5
1270715	8	2	TLE	15	5
1270747	8	2	TLE	16	5
449764	8	2	TLE	15	5
1270741	8	2	TLE	16	5
1270760	8	2	TLE	15	5
1270731	8	2	TLE	16	5
12270728	9	2	TLE	15	5
226725	8	2	TLE	17	5
226705	8	2	TLE	14	5
1270740	8	2	TLE	17	5
226780	8	2	TLE	17	5
226769	9	2	TLE	15	5
1270724	9	2	TLE	15	5
226710	9	2	TLE	15	5
1270713	8	2	TLE	14	5
226772	8	2	TLE	15	5
226756	9	2	TLE	16	5
1270725	9	2	TLE	17	5
226741	9	2	TLE	17	5
t036014	8	2	TLE	14	5
8260119	8	2	TLE	14	5
120156	9	2	TLE	16	5
468206	9	2	TLE	14	5
2191483	9	2	TLE	15	5
