# Supplementary material for: Genomic signal selection analysis reveals genes related to the lambing trait of Hotan sheep
Source: Anim Biosci. 2024 Nov 6;38(7):1384–97. doi: 10.5713/ab.24.0336 (PMC12229938; doi:10.5713/ab.24.0336)
Supplement: Supplementary file 4 [file ab-24-0336-Supplementary-4.pdf]

S4 Genes screened based on selective elimination analysis

CHROM	START	END	GENE
CM029819.1	1594244	1639224	OTOS
CM029819.1	4991450	5186449	CCDC125
CM029819.1	13064187	13146410	C1Q
CM029819.1	26288829	26457311	OSBPL9
CM029819.1	26458649	26570138	GP41
CM029819.1	35305900	35586112	DUF5580
CM029819.1	46706557	46706691	TMSB4
CM029819.1	63888150	63917745	BCAP
CM029819.1	66772196	66860116	GBP1
CM029819.1	66872096	66889462	GBP1
CM029819.1	66897782	66974077	GBP5
CM029819.1	66981720	67095889	GBP
CM029819.1	67131845	67132825	ATAC1
CM029819.1	67145125	67195437	COIL
CM029819.1	67224616	67254326	GBP
CM029819.1	67284355	67302306	Bin3
CM029819.1	71974918	72049422	ABCD3
CM029819.1	76424946	76552501	PIK3C2
CM029819.1	77102498	77153878	PAP2
CM029819.1	110050369	110733753	CD1
CM029819.1	114407282	114462905	FCGR2C
CM029819.1	122074066	122340382	Got1
CM029819.1	122554712	122629005	LAD1
CM029819.1	122857540	122915775	CLIC6
CM029819.1	122979388	123101048	RCAN1
CM029819.1	123179357	123179680	RCAN1
CM029819.1	123227715	123247342	KCNE2
CM029819.1	129712400	129712915	NAP1L1
CM029819.1	129712948	129745532	NAP1L1
CM029819.1	145198878	145354742	ABCC13
CM029819.1	145354899	145388110	LIPI
CM029819.1	187787749	187830611	HGD
CM029819.1	187839513	187876008	RABL3
CM029819.1	187880882	187909961	TFIIE1
CM029819.1	234076718	234148877	GMPS
CM029819.1	249545055	249758539	RASA2
CM029819.1	266435208	266526196	TRAPPC10
CM029819.1	266529861	266540419	GATD3
CM029820.1	26293947	26481889	AUH

CM029820.1	29874713	29999822	SLC35D2
CM029820.1	41367139	41445078	ADAM28
CM029820.1	42916161	43137220	PEBP4
CM029820.1	49863915	49887114	ANP32B
CM029820.1	49927333	49938090	HEMGN
CM029820.1	49941592	49952001	TRMO
CM029820.1	59984867	59986141	FOXB2
CM029820.1	60092218	60381172	PRUNE2
CM029820.1	86267779	86490390	SH3GL
CM029820.1	105534514	105553372	BLK
CM029820.1	106556923	106557507	GST
CM029820.1	106569433	106731156	CEP44
CM029820.1	130734193	131205890	ZNF385B
CM029820.1	175299582	175302637	CXCR4
CM029820.1	179206186	179207115	GPR39
CM029820.1	187187891	187421260	CLASP1
CM029820.1	205209858	205354768	BMPR2
CM029820.1	205406119	205475675	FAM117B
CM029820.1	205475738	205486024	BZW1
CM029820.1	205487142	205576753	ICA1L
CM029820.1	209412472	209459880	FASTKD2
CM029820.1	220441532	220702246	TNS1
CM029820.1	225341397	225410774	FARSB
CM029820.1	225429553	225431331	GK
CM029820.1	225433159	225463158	MOGAT1
CM029820.1	237258910	237259242	TLE
CM029820.1	237259288	237322389	MATN1
CM029821.1	10751653	10755362	HSPA5
CM029821.1	10756955	10785634	RABEPK
CM029821.1	10786008	10820904	RABEPK
CM029821.1	18201772	18202490	ID2
CM029821.1	18228523	18312688	ARMS
CM029821.1	36058868	36797427	ALK
CM029821.1	46635556	46673523	XPO1
CM029821.1	57982716	58066929	RPA1
CM029821.1	58067388	58094801	PTCD3
CM029821.1	58100890	58304807	IMMT
CM029821.1	70192156	70299763	PSME4
CM029821.1	70325413	70348057	ERLEC1
CM029821.1	91995090	92137193	MEMO1
CM029821.1	97298214	97299236	EI24
CM029821.1	97383970	97396819	FHL2

CM029821.1	99384451	99653235	SLC9A4
CM029821.1	99728461	99836215	IL1R1
CM029821.1	117402934	117834568	PPFIA2
CM029821.1	122111324	122234077	RASSF9
CM029821.1	131327457	131434467	FGD6
CM029821.1	131434806	131511354	VEZT
CM029821.1	138498415	138539155	PFK
CM029821.1	138540118	138591225	SENP1
CM029821.1	146783197	147123964	LRRK2
CM029821.1	173713973	173884103	CHST11
CM029821.1	196908800	197000923	EF
CM029821.1	197006930	197007595	DNAJC21
CM029821.1	197007644	197008502	DNAJC21
CM029821.1	197015583	197403214	PIK3C2
CM029821.1	201638966	201945948	GRIN2B
CM029821.1	201989084	201999576	GRIN2B
CM029821.1	202467672	202503627	KIAA1467
CM029821.1	202551768	202638956	GPRC5D
CM029821.1	216046767	216051110	NOL12
CM029821.1	216051557	216077176	TRIOBP
CM029821.1	216083028	216115922	TRIOBP
CM029821.1	216145310	216145894	H1F0
CM029821.1	216147816	216161000	GCAT
CM029821.1	216169927	216179162	ANKRD54
CM029821.1	216180159	216211629	EIF3L
CM029821.1	218553907	218593864	SLC25A17
CM029821.1	218604449	218627805	ST13
CM029821.1	218628442	218670089	XPNPEP3
CM029821.1	218677406	218689547	RBX1
CM029822.1	12554894	12621978	SGCE
CM029822.1	22833537	22927544	ETV1
CM029822.1	49635252	49916410	COG5
CM029822.1	49921908	49931766	DUS4L
CM029822.1	49935099	49981500	BCAP29
CM029822.1	62209209	62261512	ANLN
CM029822.1	62282769	62336899	KIAA0895
CM029822.1	103351403	103463400	TRIM24
CM029823.1	2008079	2134813	ADAMTS2
CM029823.1	12120739	12121752	OR7E24
CM029823.1	12138639	12139175	OR7E24
CM029823.1	12145818	12146648	Olfr18
CM029823.1	19833990	20027361	RAD50

CM029823.1	20058019	20064693	IRF1
CM029823.1	20943889	21183753	RAPGEF6
CM029823.1	21234716	21254821	RRM1
CM029823.1	32415839	32521302	HSD17B4
CM029823.1	39352289	39353236	OR2G2
CM029823.1	39377498	39378457	Olfr303
CM029823.1	39420039	39421007	CHRM2
CM029823.1	39458253	39459224	OR11L1
CM029823.1	39470805	39484182	TRIM58
CM029823.1	39496431	39497402	OR2W3
CM029823.1	60517168	60585543	ANXA6
CM029823.1	60598374	60632486	CCDC69
CM029823.1	77289245	78266293	TENM2
CM029823.1	79941484	79941744	HMGB2
CM029824.1	25079359	25081829	DDIT4L
CM029824.1	25114862	25253303	ARTD15
CM029824.1	25346762	25356772	LAMTOR3
CM029824.1	25358072	25508147	DAPP1
CM029824.1	26711906	26914860	TSPAN5
CM029824.1	29828897	30218551	UNC5
CM029824.1	58071933	58107618	PGM2
CM029824.1	87307191	87804218	SLC4A4
CM029825.1	12852744	13156948	MEGF11
CM029825.1	34524973	34581715	THBS1
CM029825.1	34586387	34923224	FSIP1
CM029825.1	35113922	35185615	BUB1B
CM029825.1	35223768	35234673	PAK6
CM029825.1	35239881	35241029	ANKRD63
CM029825.1	35247441	35268324	PLCB
CM029825.1	35281730	35282188	INAFM2
CM029825.1	35292823	35298381	CCDC9B
CM029825.1	51847622	51854587	POLR2M
CM029825.1	51873718	51987717	MYZAP
CM029825.1	54931064	55444833	UNC13A
CM029825.1	55594377	55597319	UNC13A
CM029825.1	55637373	56069936	WDR72
CM029825.1	61921903	61953400	SEMA6
CM029825.1	74690592	74784287	RHOJ
CM029826.1	10202291	10298897	UBE3D
CM029826.1	10303788	10418529	DOPEY1
CM029826.1	29386170	29434266	SNX3_12
CM029826.1	36979440	37039028	SIM1

CM029826.1	48659247	48894841	RNGTT
CM029826.1	52861663	52897189	MRAP2
CM029826.1	60391375	60392163	SPIN1
CM029826.1	60426833	60453259	ALDH8A1
CM029826.1	60462379	60549564	HBS1
CM029826.1	73932624	73972981	MAP3K7IP2
CM029826.1	75703326	75794570	CCDC170
CM029827.1	27885733	27891500	TRIB1
CM029827.1	44358253	44487815	ARFGEF
CM029827.1	44488551	44507572	ECD
CM029827.1	75264199	75264537	ELOC
CM029828.1	26038851	26710825	NBEA
CM029828.1	27280679	27280984	LRR_8
CM029828.1	27319239	27398310	ANT
CM029828.1	30777444	30811795	KATNAL1
CM029828.1	36765268	37001211	RNF17
CM029828.1	75425644	75660935	ABCC4
CM029828.1	80517207	80720841	UBAC2
CM029828.1	80731894	80732874	Gpr183
CM029828.1	90639242	90654811	SPACA7
CM029829.1	24380299	24427090	SP2
CM029829.1	24434182	24440633	pdxH
CM029829.1	24446474	24446776	PRR15L
CM029829.1	24462927	24480875	CDK5RAP3
CM029829.1	24507857	24517318	COPZ2
CM029829.1	24529156	24537606	NFE2L1
CM029829.1	24546528	24550590	CBX1
CM029829.1	24598526	24901872	SKAP1
CM029829.1	25466696	25570934	B4GALNT2
CM029829.1	25598290	25598960	GNGT2
CM029829.1	25601952	25610771	ABI3
CM029829.1	25612292	25613041	PHOSPHO1
CM029829.1	25618027	25618278	DENN
CM029829.1	62094844	62095449	MIS12
CM029829.1	62129077	62371875	CA10
CM029830.1	4678992	4686039	DYRK3
CM029830.1	4722842	4767302	MAPKAPK2
CM029830.1	18618143	18681334	KCTD3
CM029830.1	35170891	35195284	E4
CM029830.1	36186775	36222356	DPT
CM029830.1	48512652	48584823	AJAP1
CM029830.1	54413768	54417180	PRAMEF12

CM029830.1	54478588	54529367	PRAMEF12
CM029831.1	810802	1547764	PH
CM029831.1	3388158	3460716	SNAP25
CM029831.1	17177946	17261050	ABI1
CM029831.1	17263111	17302699	PDSS1
CM029831.1	22161580	22372897	MLLT10
CM029831.1	25749485	26057707	MYO3
CM029831.1	31180675	31322887	ST8SIA6
CM029831.1	47435392	47495538	GPCPD1
CM029831.1	47585025	47746165	C20orf196
CM029832.1	25790450	25830576	NDRG4
CM029832.1	25833834	25837110	SETD6
CM029832.1	25838924	25894977	CNOT1
CM029832.1	25964186	25973546	SLC38A7
CM029832.1	25981120	26002161	GOT2
CM029832.1	26037124	26037441	PPIA
CM029833.1	2698032	3018720	CASP4
CM029833.1	20408947	20590669	ARHGAP20
CM029833.1	29452102	29459713	CCDC84
CM029833.1	29459980	29461690	RPS25
CM029833.1	29462192	29465237	TRAPPC4
CM029833.1	29466689	29471266	SLC37A4
CM029833.1	29477995	29478252	C11orf58
CM029833.1	29479334	29513745	HYOU1
CM029833.1	29515354	29534138	VPS11
CM029833.1	29537793	29544692	HMBS
CM029833.1	29546054	29546485	H2A
CM029833.1	29547773	29551969	ALG7
CM029833.1	29557019	29565715	C2CD2L
CM029833.1	29574557	29580173	HINFP
CM029833.1	29596947	29608804	ABCG4
CM029833.1	36041725	36420894	SOX6
CM029833.1	77304785	77305702	OR4C6
CM029833.1	77322000	77322917	PDZ
CM029833.1	77339672	77340598	OR4C6
CM029833.1	77357843	77358769	OR4C6
CM029833.1	77381383	77382312	OR4C6
CM029834.1	10205263	10229213	SMN1
CM029834.1	10238837	10362745	NAIP
CM029834.1	22288076	22459067	MIER3
CM029834.1	22468479	22476158	SETD9
CM029834.1	33594346	33668967	MROH2B

CM029834.1	33691384	33747545	C7
CM029834.1	35321420	35491766	FYB
CM029834.1	35519115	35638600	RICTOR
CM029834.1	36764600	36793358	GDNF
CM029834.1	36836677	37113274	WDR70
CM029834.1	56768780	56925926	FAM134B
CM029834.1	56934859	56948455	ZNF622
CM029834.1	61136430	61137329	DPPA4
CM029835.1	13960362	14069512	HHIP
CM029835.1	68017209	68423803	TTC28
CM029836.1	26052871	26063480	TMED3
CM029836.1	31126984	31142830	IDH3
CM029836.1	31144526	31204605	ACSBG
CM029836.1	41725471	41725962	RRP36
CM029836.1	43063313	43120698	ARHGAP5
CM029837.1	13547088	13547591	DNAJA1
CM029837.1	13547723	13548058	DNAJA1
CM029837.1	13570678	13634867	YHHN
CM029837.1	28888261	28986422	SHQ1
CM029838.1	3162859	3258000	ZNF451
CM029838.1	3282115	3282561	KIAA1586
CM029838.1	11355328	11405739	CMTR1
CM029838.1	11408338	11423235	CCDC167
CM029838.1	11486500	11638644	MDGA1
CM029838.1	11994839	12071669	ZFAND3
CM029838.1	13775646	13826869	DAAM
CM029838.1	13831012	13864115	MOCS1
CM029838.1	26033262	26033927	RPS2
CM029838.1	30901335	30944233	PRSS16
CM029838.1	30944565	30944945	H2B
CM029838.1	30945187	30954105	H4
CM029838.1	30954426	31001507	H2B
CM029839.1	18102489	18179478	PAK1
CM029839.1	18429868	18755933	GDPD4
CM029839.1	33847627	34077312	NTM
CM029839.1	41298338	41305889	SLC22A6
CM029839.1	41315939	41336071	SLC22A8
CM029839.1	41343406	41391157	SLC22A10
CM029839.1	50860110	50871405	B4GALNT4
CM029839.1	50929853	50930706	IFITM3
CM029839.1	50933536	50968298	IFITM3
CM029839.1	50984309	50985353	IFITM5

CM029839.1	50988510	50992106	ATHL1
CM029839.1	50995971	51001330	NLRP6
CM029840.1	6719047	7675273	PRKG1
CM029840.1	22173347	22412533	FBXW
CM029840.1	22440062	22445006	POLL
CM029841.1	13158017	13165497	SYT4
CM029841.1	49242193	49269211	SMAD7
CM029841.1	49364146	49493731	DYM
CM029842.1	12046957	12081663	ATF7IP2
CM029842.1	12122081	12130438	EMP2
CM029843.1	6075933	6145578	KCNK1
CM029843.1	32956032	33074873	DLG5
CM029843.1	33110615	33154115	RPC1
CM029845.1	17132249	17132625	RPL31
CM029845.1	17201784	17202239	ARL14EPL
CM029845.1	20105758	20372779	PHEX
CM029845.1	21569547	21621419	PTCHD1
CM029845.1	48904573	48904871	MED31
CM029845.1	48934027	48934233	FA58C
CM029845.1	56573381	56584733	SUV39H
CM029845.1	56589457	56596445	WAS
CM029845.1	56644855	56650960	WDR13
CM029845.1	56664847	56666928	RBM3
CM029845.1	56679518	56691982	TBC1D25
CM029845.1	56695149	56697718	EBP
CM029845.1	82366236	82373434	SPRY3
CM029845.1	85711914	85769187	CD99L2
CM029845.1	85772157	85833979	MTMR
CM029845.1	85841444	85906834	GRAM
CM029845.1	89695028	89695601	FMR1NB
CM029845.1	89704004	89704577	FMR1
CM029845.1	89989493	89991007	SLITRK2
CM029845.1	98581533	98614108	ZIC3
CM029845.1	101655220	101656191	CCDC160
CM029845.1	117819308	117939259	KLHL9_13
CM029845.1	123258703	123259164	ZCCHC16
CM029845.1	123291455	123291916	ZCCHC16
CM029845.1	123330089	123330550	ZCCHC16
CM029845.1	124225603	124304654	ALG13
CM029845.1	125492013	125628401	CHRDL1
CM029845.1	126756410	126881202	GUCY2F
